# Photo-selective shading screens as a cover for production of purple lettuce

**DOI:** 10.1038/s41598-021-94437-5

**Published:** 2021-07-22

**Authors:** Ramon Amaro de Sales, Evandro Chaves de Oliveira, Eduardo Buzatto, Robson Ferreira de Almeida, Marcus José Alves de Lima, Sávio da Silva Berilli, Ronilda Lana Aguiar, Mario Lovo, Robson Prucoli Posse, Joana Casagrande dos Santos, Waylson Zancanella Quartezani, Rodrigo Amaro de Salles, Felipe Cunha Siman

**Affiliations:** 1 Departament of Plant Science, Federal University of Viçosa, Viçosa, Minas Gerais 36570-900 Brazil; 2 Federal Institute of Espírito Santo-Unit Itapina, Highway BR-259, Km 70, Country Side, Post office box 256, Colatina, Espírito Santo 29709-910 Brazil; 3 Federal Rural University of the Amazon, Rua Professora Antônia Cunha, Capitão Poço, Pará 68650-000 Brazil; 4 Federal University of Espírito Santo-Unit Alegre, Alto Universitário, s/n, Guararema, Alegre, Espírito Santo 29500-000 Brazil

**Keywords:** Climate change, Sustainability

## Abstract

Photo-selective shading screens are emerging practices that aim to combine crop physical protection with different solar radiation filtration to achieve desired physiological responses. The objective of the present study was to evaluate the effect of photo-selective shading screens on the growth and physiology of purple lettuce for two transplanting seasons in tropical climate in Brazil. A 2 × 4 factorial arrangement was used, being considered the first factor, the transplanting season (spring and fall), and the second factor is the three 35% shading photo-selective shading screens (red, black and silver) and full sun control. The experimental design was a randomized complete block with four replications. The variables studied were: total fresh matter, total dry matter, leaf number, stem diameter, stem length, leaf area, SPAD chlorophyll index, nitrogen balance indexes, chlorophyll, flavonoids and anthocyanins. The photo-selective shading screens influenced the microclimate and the growth variables of purple lettuce, mainly when these plants were sown in the fall. Planting lettuce during spring may result in lower yields due to the higher investment of plants in secondary metabolites to defend against abiotic stress. According to the results, photo-selective shading screens are an appropriate agronomic technique to reduce phenolic compounds and improve lettuce cultivation conditions and can be implemented within protected cultivation practices to improve crop performance.

## Introduction

World vegetable production has increased considerably over the past five decades, in which population growth plays a key role in the growing demand for these foods^1^. Among these vegetables, lettuce (*Lactuca sativa* L.) has been gaining more and more market share, where in Brazil it is among the most important cultivated vegetables.

Lettuce is a leafy vegetable, excellent source of minerals, fibers and bioactive compounds, such as folate (vitamin B9), β-carotene, lutein and phenolics that are beneficial to human health^2^, being generally consumed fresh in salad mixes with other fresh vegetables. Global lettuce production in 2017 was approximately 27 million tons^3^. In 2020, Brazil produced about 1.5 million tons of lettuce and had a per capita consumption of 8.6 kg/inhabitant/year^4^.

The main cultivation techniques for vegetables in Brazil are those of open field cultivation, protected environment and hydroponic cultivation, being used according to the technical level of the producer and the need to maximize production. In this context, cultivation in protected environments has revolutionized the production of vegetables, making it possible to condition the microclimate to the needs of plants and extending the production period to times of the year and regions previously unfit for certain crops^5^.

The black shading network is one of the materials most widely used, however, pigmented networks (photo-selective) are increasingly being used in commercial production^6^. The use of pigmented screens, or technically photo-selective screens, is recent in Brazil and its effects go beyond isolated temperature interference. They can increase the relative proportion of diffused light and absorb several spectral bands, thus affecting the quality of light^7,8^. Holcman and Sentelhas^9^, found that the type and color of the shading meshes affect the microclimate of the environment, mainly the intensity and quality of solar radiation.

The fraction of light that passes through the orifices of the photo-selective screens remains unchanged in quality, while the light that reaches the wires is spectrally modified and scattered at the outlet^10^. Therefore, the combination of light scattering and spectral manipulation can modify desirable characteristics in plants^11^.

This technique is spreading rapidly in protected horticulture^12,13^, however, its effects on the yield and secondary metabolism of vegetables, such as flavonoids and anthocyanins, are still uncertain.

Variations in light intensity between 400 and 600 μmol m^−2^ s^−1^ represent optimal values for growing lettuce, depending on its latitude^14^. However, in most regions of Brazil this value is exceeded, making it necessary to reduce the impacts of the high intensity of irradiance. As noted by Ilić et al.^15^, technologies such as photo-selective screens for shading are emerging practices, which aim to combine the physical protection of cultures with different filtrations of solar radiation.

Evaluating the effect of the red photo-selective screen on saline pepper plants (*Capsicum annuum* L.) in Spain, the authors Gálvez et al.^16^ demonstrate that shading is an efficient management strategy to modulate microclimate conditions at the crop level, controlling ionic homeostasis and the hormonal balance of the plant to deal with salt stress. Preliminary results showed that the use of red photoelectric screens significantly increased the productivity of the zucchini^17^.

The use of photo-selective screens, therefore, becomes a technique capable of bringing important results in tropical agriculture, increasing the productivity of crops, as a result of physiological and biochemical changes. In this context, the objective of this research was to evaluate the effects of the planting season and of photo-selective nets for the Mimosa Roxa Salad Bowel variety on growth, yield and physiology in tropical climate in Brazil.

## Materials and methods

The experiment was carried out in the experimental area of the Federal Institute of Education, Science and Technology of Espírito Santo—Itapina Campus, located in Colatina, Espírito Santo state, southeastern Brazil, with geographic coordinates of 19° 32′ 22″ S, 40° 37′ 50″ W, elevation of 71 m. The climate of the region is Tropical Aw, according to the Köppen climate classification, with a well-defined rainy season between October and January, high temperatures and 1029.9 mm average rainfall^18^.

Lettuce was sown in a 345-cell Green-Up phenolic foam substrate, produced in a greenhouse by hydroponic system until reaching field size (25 days after sowing), with 80% of the plants presenting 4–5 definitive leaves. The NFT hydroponic system was made up of four 3 m long benches, containing 12 polypropylene profiles, with 75 mm of diameter, spaced at 25 cm between each profiles.

Lettuce, cultivar Mimosa Roxa Salad Bowel, was grown in phenolic foam, irrigated for five days with pure water. The cells were then detached and transplanted to the nursery, receiving standard nutrient solution from the Horticulture Sector of the Federal Institute of Education, Science and Technology of Espírito Santo, Itapina Campus, adapted by Cometti et al.^19^ with EC = 1.0 dS m^−1^.

The soil of the experimental area is classified as Dystrophic Red–Yellow Latosol^20^. Soil samples were collected at random points from the experimental area of 0.5 hectares, for soil analysis and recommendation for fertilization according to Prezotti et al.^21^. Deep plowing and two harrowing were done to break the clods and homogenize the soil, facilitating the construction of the beds and the planting of the seedlings.

A 2 × 4 factorial arrangement was used, the first factor considered was the season of the year for planting, and the second factor the type of cover, being three different photo-selective screens and a control planting in full sun. The experimental design was a randomized complete block with four replications, and the experimental unit consisted of beds of 2 m in length and 1.0 m in width, with four planting lines each, with a useful area composed of two central lines, disregarding the surround lines.

Thus, two plantings were carried out in different seasons, where one was planted in the spring (18/09/2017) and the second planting in October (17/04/2018). In the composition of the treatments, three artificially generated light environments were generated by using different photo-selective shading screens (35% shading index), aiming to create environments with different light qualities, namely: T-Red, constituted red sombrite; T-Black, consisting of black sombrite; T-Silver, consisting of silver-colored sombrite and T-Control, which were plants grown in the open.

The low tunnel had 1.5 m of height and 1.20 m of base. The sides were kept open at a height of 0.30 m from the base to allow air circulation. Both tillage systems were covered in black and silver mulching, to avoid weeds growth. The temperatures and luminosity of the environments, during the experimental period, were recorded in a Data Logger Hobo apparatus placed at the canopy height of the plants, adjusted according to the plant’s growth.

At the end of the experiments, 30 days after transplanting in both seasons (Spring and Autumn), the chlorophyll indices, nitrogen balance and secondary compounds were evaluated with colorimetry equipment (SPAD 502—Minolta) and a fluorescence sensor (FORCE-A, Orsay, France) which are without unit indexes. The analyzes were performed in the morning, between 9:00 and 11:00 h and on only one side of the seedlings, pointing the equipment to the crown, from the top to the bottom, at an angle of approximately 45°.

The Multiplex^®^ (FORCE-A, Orsay, France) is a hand-held, multi-parametric fluorescence sensor that uses LED excitation light and filtered-photodiode detection. The Multiplex^®^ indices were calculated by four excitation wavelengths: ultraviolet (UV, 373 nm), blue (B, 470 nm), green (G, 516 nm), Red–orange (R, 635 nm); and three detection wavelengths: yellow (YF, 590 nm), red (RF, 685 nm), and far-red (FRF, 735 nm), estimating indices of various compounds, such as nitrogen balance (NBIG and NBIR), total chlorophyll (SFRG and SFRR), anthocyanin (ANTRG and ANTRB) and flavonoids (FLAV)^22,23^.

After the vegetables were harvested, the plants were immediately transferred to the laboratory of the Federal Institute of Education, Science and Technology of Espírito Santo—Campus Itapina, to evaluate the following characteristics: number of leaves (leaves greater than 0.01 m), stem length and diameter (with the aid of a caliper), total leaf area (using LI-3100 Area Meter leaf area meter), fresh and dry phytomass (measurements made with precision analytical balance). Dry weight was evaluated after drying the plants in a circulating air oven at 70 °C to constant weight.

Prior to the analysis of variance, it was verified the normality of errors and homogeneity of variance by using Shapiro–Wilk and Bartlet tests, respectively. Complying the assumptions after the verification, the analysis of variance was performed by the F test and when significant submitted to the Tukey test at a significance level of 5% probability (p < 0.05). To determine the influence of photo-selective screens, multivariate analysis using the main components was also performed. Statistical analyzes were performed using the open source program R.

## Results and discussion

### Microclimate conditions

**Figure 1:**
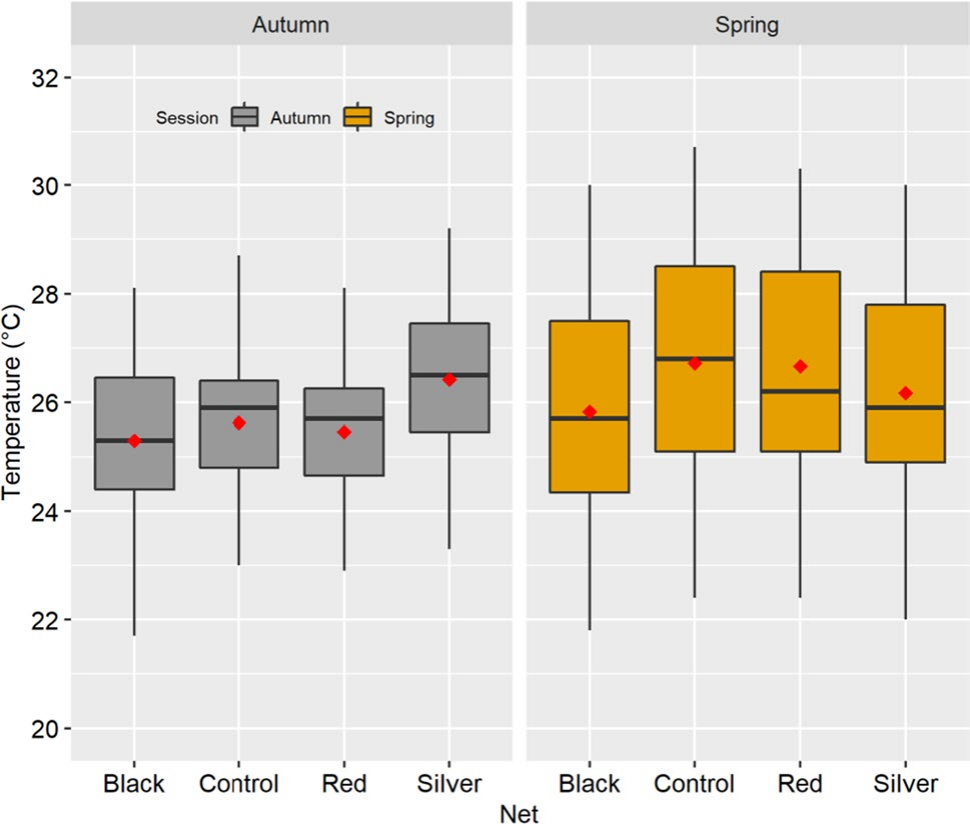
The average temperature variability within the photo-selective screen structures during the cultivation period (30 days) of the lettuce in the autumn and spring seasons.

The graphical analysis showed that the microclimatic conditions of each environment, in which the lettuce was developed, apparently did not undergo major changes, judging by the variability of the average daily temperature (Adt) inside the structures of photo-selective screens and in the open field (control treatment), indicating that there is no statistical difference in Adt between the treatments analyzed (Fig. 1).

However, it was observed that the average temperature in the control treatment, during the lettuce cycle (Atc), both in autumn and spring, were 25.6 °C and 26.7 °C, respectively, therefore a difference of 1.1 °C. On the other hand, observing the 75% percentile, it was observed that in the autumn productive cycle 75% of the days presented Adt equal to or less than 26.5 °C whereas in the spring this value was 28.6 °C (Fig. 1), certainly indicating the occurrence of the warmest period in spring.

The use of photo-selective screens, in autumn cultivation, practically did not change the Adt inside the structures, with the exception of the silver screen, which apparently conserved the heat, presenting a Adt superior to the control treatment (Fig. 1). On the other hand, the use of the screens in the spring promoted reductions in Adt, of up to 1.9 °C, 1.1 °C and 1.3 °C for the black, red and silver screens, respectively, when compared to the control treatment.

Although photo-selective screens did not provide a statistically significant reduction in Adt, they promoted a high frequency of days with Adt below Atc in the open. For example, during the autumn production cycle, black, red and silver fabrics promoted 55%, 48% and 29% of the days with Adt below Atc, while the same screens in the spring planting promoted 65%, 52%, 58%, respectively, indicating lower temperatures on at least 29% of the days in the fall and 52% in the spring.

The silver screen exhibited an interesting feature, in microclimate terms, it conserved the heat in the autumn period and reduced in the spring. This type of material has great potential for use, especially in regions with large variations in Adt, between southern stations.

### Analysis of variance and effect of planting season

The analysis of variance in Table 1 reveals that there is no significant interaction between the factors under study (season × screen) for all evaluated characteristics, in purple lettuce. Therefore, only the simple effects of the factors were analyzed, within the season in which they were planted and the effect of the types of cover used (photo-selective screen) on the growth and physiology variables. When factors were analyzed in isolation, significant effects were observed, both in growth and physiological variables, indicating considerable microclimate changes, although in absolute and statistical terms they seemed insignificant.

**Table 1:** Summary of analysis of variance of morphological and physiological characteristics.

Source of variance	DF	Mean squares (MS)						
		TFM	TDM	NL	SD	SL	LA^a^	SPAD
Season	1	30,381.1**	12.90*	153.5*	439.47**	12,394.2**	3262.6**	222.65*
Error a	4	243.7	1.124	1.75	0.81	108.4	28.61	4.39
Screen	3	1507.0*	3.376*	2.37^ns^	0.86^ns^	1341.1**	189.4*	32.85**
Season × screen	3	221.9^ns^	0.774^ns^	9.57^ns^	1.14^ns^	219.4^ns^	45.8^ ns^	7.31^ns^
Error b	12	393.4	0.765	5.16	2.89	105.5	50.98	3.75

Source of variance	DF	SFRG	SFRR	NBIG	NBIR	FLAV	ANTHRG	ANTHRB
Season	1	0.870**	1.016 **	4.05**	2.35**	1.174**	0.00806^ns^	0.00007^ns^
Error a	4	0.0208	0.00067	0.0128	0.0117	0.00032	0.0009	0.00033
Screen	3	0.103*	0.0213*	0.0138^ns^	0.0004^ns^	0.018**	0.00711*	0.0061*
Season × screen	3	0.054^ns^	0.0099^ns^	0.0218^ns^	0.019^ns^	0.0013^ns^	0.00450^ns^	0.0018^ns^
Error b	12	0.0197	0.0049	0.0157	0.0205	0.0004	0.00152	0.00107

*TFM* total fresh matter (g), *TDM* total dry matter (g), *NL* number of leaves, *SD* stem diameter (mm), *SL* stem length (mm), *LA* leaf area (cm^2^).

*Significant at 5% of probability (p < 0.05).

** Significant at 1% of probability (p < 0.01); *ns* no significant.

^a^Values divided by 10^3^.

For example, the microclimate variability modulated by the sowing dates during the productive cycles of autumn and spring, promoted significant effects at the level of 1% probability in all biometric and plant growth variables such as dry biomass. In addition, there were effects at the biochemical level, such as the biosynthesis of chlorophyll, flavonoids and anthocyanins, evidencing an expected fact, since the sowing dates are in different austral seasons and at a latitude of 19.5° S. Therefore, under conditions optimum water levels in the soil, such variations are modulated by crop genetics and microclimate variability, especially solar radiation and air temperature.

Regarding the types of coverage, that is, photo-selective screens (Table 1), only the variables NL and SD and NBIG and NBIR did not have a significant effect, suggesting that the use of photo-selective screens provides qualitative gains and acceleration of parameters growth. Such results suggest that the statistical characterization of the environment, in terms of frequency, is much more interesting than the application of averages tests, when the objective is to evaluate microclimate interactions with plant growth. According to Ntsoane et al.^6^, changes in the quality of light can potentially alter the physiological, biochemical processes and, consequently, the growth, development, production and quality of plants.

Through the analysis of the Table 2 content, it is possible to observe the effect of sowing season on lettuce growth and yield. There was higher total fresh matter production, total dry matter, plant height, stem diameter, stem length and leaf area when these plants were sown in autumn. As noted by Silva et al.^24^ evaluating different lettuce cultivars (smooth, curly and purple) during summer, autumn and winter, the authors found higher yield values for all cultivars when produced in autumn in the Sergipe region, Brazil. This is the most appropriate period for cultivation according to the authors in that region.

**Table 2:** Mean values of total fresh and dry matter (MPT and MDT in g), number of leaves (NL), stem diameter (SD in mm), stem length (SL in mm), leaf area (LA in cm^2^) and leaf chlorophyll index (SPAD) of purple lettuce grown in different seasons of the year.

Seasons	TFM	TDM	NL	SD	SL	LA	SPAD
Spring	91.9 b	5.6 b	13.9 b	12.5 b	67.8 b	1005.6 b	26.6 a
Autumn	163.0 a	7.1 a	19.0 a	21.0 a	113.3 a	1742.9 a	20.5 b
CV%	12.24	16.71	8.01	5.36	11.49	12.31	8.89

Means followed by the same letter do not differ from each other at the 5% probability level (p < 0.05) by the Tukey test.

High temperatures are common in the most of the Brazilian territory, being a limiting factor for the production of lettuce and vegetables in general, as observed during spring. When lettuces are subjected to high temperatures, there is an accelerated inflorescence formation and shortening of the vegetative period in the plants^25,26^, impairing their yield.

Vieira^27^ studying lettuces development, analyzing cv. Elisa and cv. Maíra observed that they did not exhibit satisfactory development during a spring due to high temperatures in Minas Gerais, Brazil. The use of photo-selective screens, that reduce part of the sun's incidence, were not able to allow the plantation during this period. The authors Silva et al.^28^ evaluate a leafy *Coriandrum sativum* during the winter, spring, summer, and fall growing seasons in a tropical environment, noting better development in growth variables and higher yield values for the fall season.

For the SPAD index (Table 2), which is an indirect chlorophyll meter, although it presented higher values for spring planting, this did not reflect a higher total amount of chlorophyll. Both lettuce dry yield and leaf area were higher when sown in autumn, probably because of the higher dry matter and also the larger leaf area, led to a reduction in chlorophyll concentration due to the dilution effect in reason of the higher plant growth.

This phenomenon has already been observed by several authors, such as chlorophyll leaf concentrations in orange twigs, obtained by SPAD calibration with chlorophyll extraction by Pestana et al.^29^*,* in which, according to the authors, a resumption of orange growth caused this effect. The same was observed for micronutrient Zn in cotton plants by Anwaar et al.^30^.

**Figure 2:**
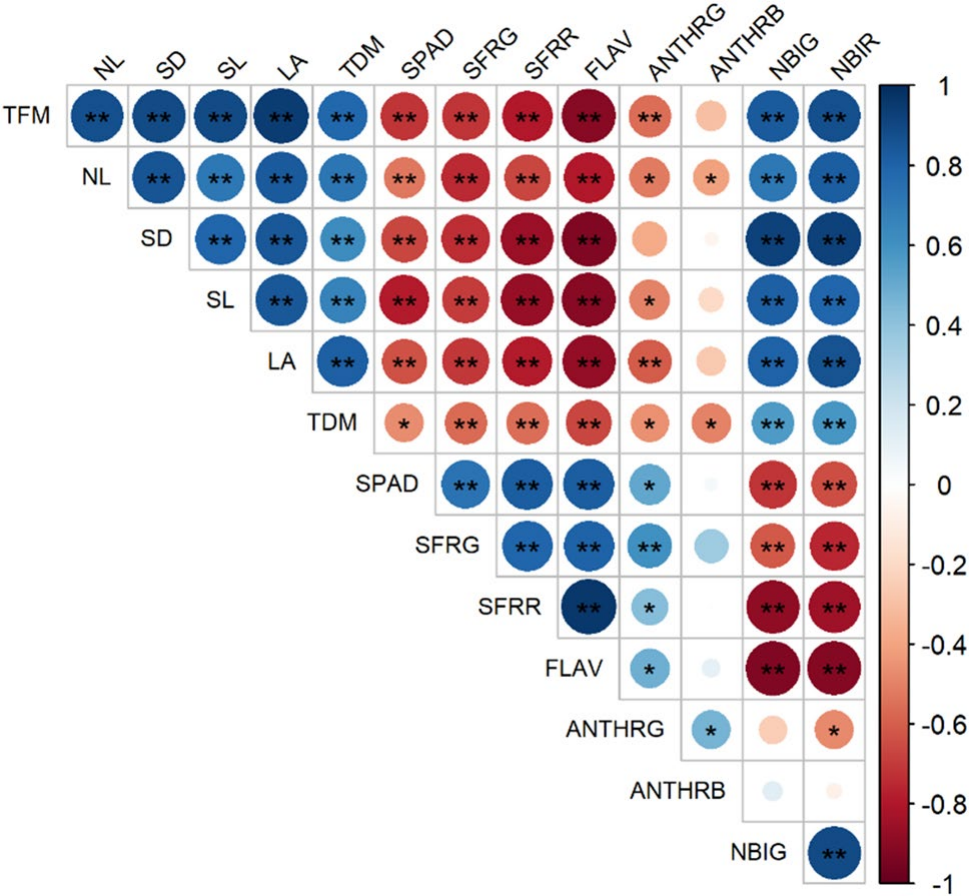
Correlation analysis between the response variables studied for purple lettuce. Significant a *p < 0.05; **p < 0.01. Positive and negative correlations are displayed in blue and red, respectively; the color intensity and circle size are proportional to the correlation coefficients. *TFM* total fresh matter, *TDM* total dry matter, *NL* number of leaves, *SD* stem diameter, *SL* stem length, *LA* leaf area, *SPAD* chlorophyll index; nitrogen balance indexes (NBIG and NBIR), chlorophyll (SFRG and SFRR), flavonoids (FLAV) and anthocyanins (ANTHRG and ANTHRB).

The sowing time provided differences for almost all variables obtained by Multiplex as observed in Table 3. It is important to highlight that the chlorophyll indices obtained by Multiplex, presented the same response pattern of SPAD, with spring planting presenting higher values of chlorophyll. By the correlation analysis (Fig. 2), it confirms the dilution effect of these chlorophyll indices, obtained by Multiplex and SPAD, by presenting negative and significant correlation with leaf expansion (LA). Thus, as plant leaf area was increasing, the plants presented lower chlorophyll concentration per unit area.

**Table 3:** Average values of nitrogen balance (NBIG and NBIR), chlorophyll (SFRG and SFRR), flavonoids (FLAV) and anthocyanins (ANTHRG and ANTHRB) indexes of purple lettuce grown at different seasons.

Seasons	NBIG	NBIR	SFRG	SFRR	FLAV	ANTHRG	ANTHRB
Spring	1.07 b	0.59 b	1.50 a	1.30 a	0.30 a	0.173 a	− 0.736 a
Autumn	1.89 a	1.22 a	1.12 b	0.89 b	− 0.14 b	0.135 a	− 0.733 a
CV%	7.61	15.05	11.00	11.39	22.99	19.37	2.48

Means followed by the same letter do not differ from each other at the 5% probability level (p < 0.05) by the Tukey test.

Nitrogen balance indices were higher during autumn planting, contributing to a greater amount of lettuce dry matter during this planting season, since N participates in amino acid and protein composition and chlorophylls. Thus, there was a noticeable increase in leaf area expansion (Table 2) during autumn and, consequently, the levels of liquid photosynthesis, resulting in greater accumulation of dry matter. As noted by Sales et al.^31^, the leaf area is responsible for capturing sunlight through its complex antenna, generating energy and directly participating in the formation of photoassimilates for the plant through photosynthesis, which will be responsible for maintenance and growth.

Although the nutrient supply was equal at both planting seasons, this higher nitrogen balance is easily explained, because during spring there was greater synthesis of flavonoids and anthocyanins (Table 3). As noted by Coelho et al.^32^ there is a competition in biosynthesis and metabolic regulation of polyphenols and proteins, which use the same precursor, the amino acid phenylalanine. Thus, due to a higher growth rate in plants grown during the autumn, protein synthesis reduced the availability of phenylalanine for use in phenolic synthesis^33^, which resulted in a lower rate of secondary compounds as anthocyanins and flavonoids, observed in this experiment.

The flavonoids and anthocyanins compounds are responsible for protecting against damage caused by UV light on leaves, which have a negative correlation with nitrogen balance (Fig. 2). Thus, vegetables accumulate a series of phenolic compounds and other antioxidants as a protective action against high irradiance and UV light^34^, which are higher during spring. The authors Sales et al.^23^ observed in the species *Schinus terebinthifolius* that a higher index of flavonoids in the leaves caused a lower nitrogen balance.

### Effect of photo-selective screens on growth and physiology

Among the variables analyzed in Table 4, the number of leaves and stem diameter did not differ between the different photo-selective screens used and the plants grown in full sun. The characteristics leaf area, fresh matter and total dry presented the same response pattern, and the red photo-selective screens, black and silver colors were statistically equal. In a study conducted by Bastías et al.^35^, it revealed that leaves under photo-selective nets have longer stomata and a greater length–width ratio compared to the control network. This may have contributed to increase photosynthesis and, consequently, larger accumulation of carbon.

**Table 4:** Average values of total fresh and dry matter (GFR and TDM in g), number of leaves (NL), stem diameter (SD in mm), stem length (SL in mm), leaf area (LA in cm^2^) and chlorophyll index (SPAD) of purple lettuce cultivated in different photo-selective screens and in full sun.

Treatments	TFM	TDM	NL	SD	SL	LA	SPAD
T-Red	141.8 a	7.3 a	16.9 a	17.2 a	98.1 a	1563.5 a	22.0 b
T-Black	134.9 ab	6.5 ab	17.1 a	16.3 a	102.5 a	1428.1 ab	22.9 b
T-Silver	127.9 ab	6.0 ab	16.2 a	16.7 a	92.6 a	1367.8 ab	22.3 b
T-Control	105.2 b	5.6 b	15.7 a	16.8 a	69.0 b	1137.7 b	27.0 a
CV%	15.56	13.79	13.76	10.12	11.34	16.42	8.22

Means followed by the same letter do not differ from each other at the 5% probability level (p < 0.05) by the Tukey test.

In contrast, the authors Tafoya et al.^36^ comparing different colors of photo-selective screens in cucumber cultivation, observed equal fruit weight in red and silver photo-selective screens, but both were larger than black photo-selective net.

There was greater stem elongation (SL) when cultivated in photo-selective screens than in full sun. As noted by Ilić et al.^37^, lower light intensities increase the elongation of the lettuce stem. Thus, the 35% shading index favored the plants, in which they presented longer stem lengths. Considering that the availability of light was similarly reduced by photo-selective screens, these results suggest that, in our study, the amount of light appeared to be more important than the quality of light for changes in growth characteristics, how suggested by Bastías et al.^35^.

The red photo-selective screen was superior to the control, planting in full sun, which provided a gain in leaf area of 37%. This greater leaf expansion allowed greater light interception, which resulted in a 30% gain in dry matter when compared to the control treatment. According to Tudela et al.^38^ in leafy vegetables the dry matter is considered as a quality parameter to guarantee the useful life. Higher lettuce weight in red photo-selective screen was observed by Ilić et al.^37^ when compared to black, blue photo-selective screens and full sun.

By covering crops with red and blue photo-selective screens, allow to achieve improvements in photosynthesis and consequently productivity, as these nets improve radiation absorption at blue and red wavelengths^39^. Red nets transmit a higher flux density of photosynthetic photons and transmit more red light within the culture environment, and ensure greater radiation scattering^34,36^. According to Sivakumar et al.^40^, the red photo-selective screens absorbs UV, blue and green (495–570 nm) and enriches the distant red and red spectral region, in which plant growth and development can be modified by changing these light levels.

For the SPAD index, it is observed that the plants cultivated in full sun (T-Control) presented the highest values (Table 4). These results can be better understood when analyzing the leaf area and dry matter, since the photo-selective screens presented higher numerical values, thus, possibly there was a dilution effect for SPAD and the chlorophyll indices obtained by Multiplex (Table 5).

**Table 5:** Mean values of nitrogen balance (NBIG and NBIR), chlorophyll (SFRG and SFRR), flavonoids (FLAV) and purple lettuce anthocyanins (ANTHRG and ANTHRB) indices grown in different photo-selective screens and in full sun.

Treatments	NBIG	NBIR	SFRG	SFRR	FLAV	ANTHRG	ANTHRB
T-Red	1.47 a	0.92 a	1.18 b	1.04 b	0.03 c	0.128 b	− 0.763 b
T-Black	1.45 a	0.91 a	1.22 ab	1.05 ab	0.04 c	0.136 b	− 0.761 b
T-Silver	1.56 a	0.91 a	1.42 a	1.14 ab	0.09 b	0.142 ab	− 0.706 a
T-Control	1.47 a	0.90 a	1.43 a	1.16 a	0.16 a	0.208 a	− 0.708 ab
CV%	8.43	15.79	10.68	6.37	20.21	26.14	4.44

Means followed by the same letter do not differ from each other at the 5% probability level (p < 0.05) by the Tukey test.

Regarding the biochemical parameters, it was observed that there was no difference for the nitrogen balance indices. Open field cultivation showed the highest levels of flavonoids, the same observed for anthocyanins, indicating that the synthesis of these compounds in lettuce produced in the open field has more expressive antioxidant activity than that of lettuce grown under shading screens.

Solar radiation and its spectral composition regulate many physiological responses in plants, including flavonoid biosynthesis, which local site of higher solar require greater protection against daily photoinhibition^11,41^, therefore, the cultivation in full sun there was greater biosynthesis and accumulation of these compounds in the epidermal tissues.

### Correlation matrix and PCA analysis

When the data were correlated to verify the association between the variables, it was observed a significant correlation (p < 0.05 and p < 0.01) in most of the studied variables, with positive and negative correlations (Fig. 2). When comparing the results of growth variables such as TFM, NL, SD, SL, LA and TDM with flavonoids, strong and negative correlations were observed. Thus, the higher the growth of these plants, the lower their investment in defense compounds. This is because the investment in defense compounds requires precursor molecules of primary metabolism^42^.

Plants allocate the resources acquired above and below the ground for their vital functions, such as growth, defense and reproduction, to maximize their suitability for the environment in which they are inserted^43^. The allocation to one function prevents its use for another, promoting trade-offs that determine resource allocation restrictions^42,44^.

These changes in resource allocation may occur because of biotic and abiotic stresses and, in the specific case of this study, by microclimate changes (abiotic) promoted by the different photo-selective screens. Therefore, such microclimate changes were able to change the allocation of resources, promoting more synthesis of defense compounds or growth.

Correlations between nitrogen balance indices (NBIG and NBIR) with growth characteristics were strong and positive according to Fig. 2. Thus, it can be stated that plants with a high nitrogen balance are in line with its growth, and it can be used as a non-destructive indicator of growth and/or biomass. According to Li et al.^45^ nitrogen balance is a very sensitive index for the diagnosis of plant N status and is, therefore, a strong indicator of N status.

**Figure 3:**
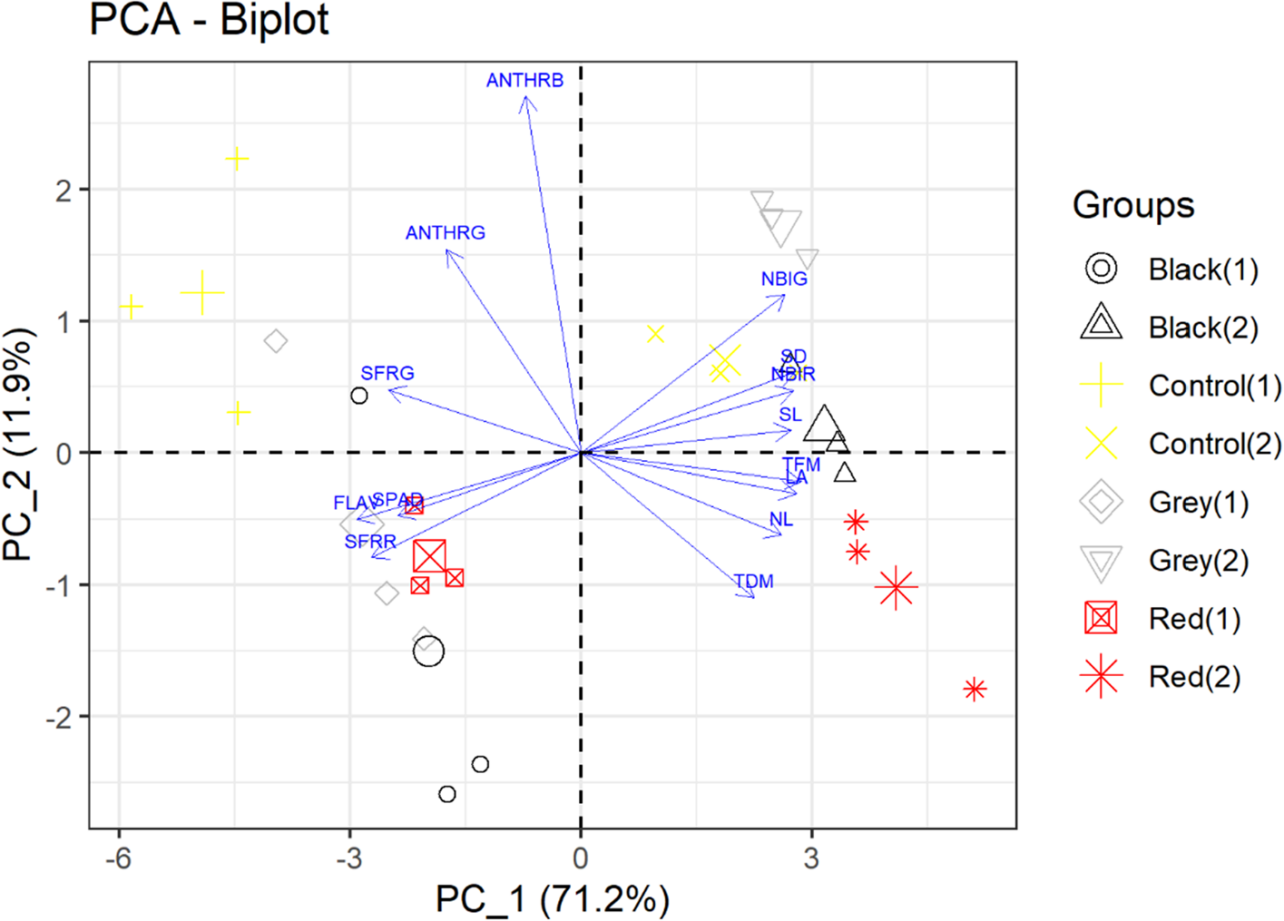
Principal component analysis of variables associated with lettuce growth and physiology cultivated two planting seasons and in different photo-selective screens (black, control (full sun), silver and red). (1) Spring; (2) Autumn. *TFM* total fresh matter, *TDM* total dry matter, *NL* number of leaves, *SD* stem diameter, *SL* stem length, *LA* leaf area, *SPAD* chlorophyll index; nitrogen balance indexes (NBIG and NBIR), chlorophyll (SFRG and SFRR), flavonoids (FLAV) and anthocyanins (ANTHRG and ANTHRB).

Figure 3 reports ordering biplotation of principal component analysis (PCA) output modeling. The first two main components reached 83.1% of the total variation, on lettuce growth and physiology, where PC_1 accounted for 71.2% and PC_2 for 11.9% of the variance of the lettuce matrix data. This allowed data variability and possible associations between variables to be described in two main axes. Thus, the characteristics that showed strong association with PC_1 and PC_2 can explain the response of plants with the different treatments used.

The variables NBIG, NBIR, SD, SL, TFM, LA, NL and TDM presented similar contributions to CP_1. These variables were strongly associated with the red, black and silver photo-selective screens grown in the autumn planting, thus contributing to the higher values in these variables. It was also observed high positive correlations between the variables SD, NBIR and SL, as they formed acute angles between the variables, the same also occurred for TFM, LA and NL.

Analyzing CP_2, it was possible to observe that the anthocyanin indices (ANTHRG and ANTHRB) presented the largest contributions. The ANTHRB index was the most important, but had little association with the photo-selective screens. The ANTHRG characteristic was more associated with the control treatment, that is, in full sun and when cultivated during spring. This is probably linked to the higher radiation availability and higher temperatures recorded during spring and full sun planting.

In general, we can see a clear separation between groups in CP_1 that presented more than 70% of the total variation, in which the characteristics that contributed the most to this component are associated with the different photo-selective screens during autumn planting. As noted by Ilić et al.^11^ shading is a technique used to control plant development and improve yield and quality before and after harvest.

Thus, despite the use of photo-selective screens aid during the spring in microclimate conditions, it presented low influence on the characteristics that contributed most in CP_1. This gives two important information, the first is about the importance of using shading screens during the fall, and the other one that the shading rate can be over than 35% during spring and summer crops in tropical regions such as Brazil. Thus, the analysis of the photo-selective meshes and planting times on the morphological and physiological characteristics is important, since it allows to group the treatments according to their growth performance.

## Conclusion

Spring lettuce crops in tropical countries may result in lower yields due to higher plant investment in secondary metabolites to defend against abiotic stresses.

Photo-selective screen appears to be an appropriate agronomic technique to reduce phenolic compounds and improve lettuce cultivation conditions and it can be implemented to improve crop performance.

The red photo-selective screens enhanced the photosynthetic activity of the plant, observed by the high gains in biomass and leaf expansion, recommended for cultivation.
